# Measurement of Total and Free Urinary Phenol and Paraben Concentrations over the Course of Pregnancy: Assessing Reliability and Contamination of Specimens in the Norwegian Mother and Child Cohort Study

**DOI:** 10.1289/ehp.1408325

**Published:** 2015-03-17

**Authors:** Virginia T. Guidry, Matthew P. Longnecker, Heidi Aase, Merete Eggesbø, Pål Zeiner, Ted Reichborn-Kjennerud, Gun P. Knudsen, Randi J. Bertelsen, Xiaoyun Ye, Antonia M. Calafat, Stephanie M. Engel

**Affiliations:** 1 Department of Epidemiology, UNC Gillings School of Global Public Health, Chapel Hill, North Carolina, USA; 2 Epidemiology Branch, National Institute of Environmental Health Sciences, National Institutes of Health, Department of Health and Human Services, Research Triangle Park, North Carolina, USA; 3 Department of Child Development and Mental Health, and; 4 Department of Genes and Environment, Norwegian Institute of Public Health, Oslo, Norway; 5 Division of Mental Health and Addiction, Oslo University Hospital, Oslo, Norway; 6 Department of Genetics, Environment and Mental Health, Norwegian Institute of Public Health, Oslo, Norway; 7 Haukeland University Hospital, Department of Occupational Medicine, Bergen, Norway; 8 Division of Laboratory Sciences, Centers for Disease Control and Prevention, Atlanta, Georgia, USA

## Abstract

**Background:**

Exposures to environmental phenols and parabens may be harmful, especially *in utero*. Prior studies have demonstrated high within-person variability of urinary concentrations across pregnancy.

**Objectives:**

We sought to measure phenol and paraben biomarker concentrations for the Norwegian Mother and Child Cohort (MoBa) study, assess within-person variability, and investigate any possible external phenol or paraben contamination of specimens.

**Methods:**

We collected three spot urine samples at approximately 17, 23, and 29 weeks gestation in a hospital setting and added a preservative containing ethyl paraben. We measured urinary concentrations and within-person variability for phenols and parabens in a MoBa sample (*n* = 45), including a subgroup of 15 participants previously randomly selected for a bisphenol A (BPA) exposure study who had unusually high total BPA concentrations. Additionally, we compared reliability results for total, conjugated, and free concentrations of phenolic compounds.

**Results:**

We detected total and free BPA, butyl paraben, propyl paraben, and methyl paraben in 100% of samples, total benzophenone-3 in 95% of samples, and infrequently detected free benzophenone-3 and total and free 2,4-dichlorophenol and 2,5-dichlorophenol. Intraclass correlation coefficients (ICCs) for total, conjugated, and free concentrations ranged from relatively low for BPA to moderate for propyl paraben. ICCs were generally similar overall and by subgroup.

**Conclusions:**

Using conjugated concentrations improved reliability estimates only for BPA. Measuring total and free concentrations, an approach that may be useful for future studies, allowed us to identify likely BPA and butyl paraben contamination of archived MoBa urine specimens.

**Citation:**

Guidry VT, Longnecker MP, Aase H, Eggesbø M, Zeiner P, Reichborn-Kjennerud T, Knudsen GP, Bertelsen RJ, Ye X, Calafat AM, Engel SM. 2015. Measurement of total and free urinary phenol and paraben concentrations over the course of pregnancy: assessing reliability and contamination of specimens in the Norwegian Mother and Child Cohort Study. Environ Health Perspect 123:705–711; http://dx.doi.org/10.1289/ehp.1408325

## Introduction

Phenols, including bisphenol A (BPA) and benzophenone-3, and parabens, which also contain a phenolic ring, are found in a number of common consumer products [Centers for Disease Control and Prevention (CDC) 2009, 2010]. The ubiquity of human exposure to phenolic compounds has sparked questions regarding toxicity, especially from exposures during sensitive periods, for example, *in utero* (Bushnik et al. 2010; Calafat et al. 2008, 2010; Mahalingaiah et al. 2008; Vandenberg et al. 2010; Wolff et al. 2008; Woodruff et al. 2011). Prenatal exposures to phenols have been associated with decreased gestational age at birth (BPA and benzophenone-3) (Tang et al. 2013), decreased birth weight (2,4-dichlorophenol and 2,5-dichlorophenol) (Philippat et al. 2013; Wolff et al. 2008), and child behavior problems, with some evidence of sex differences (BPA) (Braun et al. 2009, 2011b; Harley et al. 2013; Perera et al. 2012). Concentrations of phenol and paraben biomarkers in spot urine samples, which provide exposure measurements at one point in time, are frequently used to estimate recent exposures. However, many phenolic compounds are rapidly excreted (Völkel et al. 2002), and the degree to which a single measure can be used to represent longer-term or cumulative exposure depends partly on population-specific factors such as geographic location, demographic characteristics, and lifestyle (Koch et al. 2014; Meeker et al. 2013; Ye et al. 2009).

Phenolic compounds in humans can be metabolized via conjugation [Matthews et al. 2001; U.S. Environmental Protection Agency (EPA) 2002]; these conjugated species are readily excreted via urine (Völkel et al. 2002). Generally, conjugated phenolic compounds comprise the bulk (≥ 90%) of the total biomarker concentration in urine (Koch et al. 2012; Liao and Kannan 2012; U.S. EPA 2002; Völkel et al. 2002; Ye et al. 2005b). The remainder of the total urinary phenolic biomarker concentration consists of unconjugated (free) species that can arise from several sources: *a*) free phenolic compounds that passed through the body without conjugation, representing a biologically active form (Koch et al. 2012; U.S. EPA 2002; Völkel et al. 2002); *b*) free phenolic compounds that entered the sample via contamination during collection or processing (Koch et al. 2012; U.S. EPA 2002; Völkel et al. 2002); *c*) conjugated phenolic compounds hydrolyzed to the free form by enzymes in local tissues (Waechter et al. 2007); or *d*) conjugated phenolic compounds hydrolyzed to the free form following improper storage or handling (Ye et al. 2007).

In field studies, exposure assessment is complicated by the ubiquitous presence of phenolic compounds in the environment. This has implications for population exposure levels as well as protocols for sample collection, processing, and analysis, which must be designed to minimize the potential for external sample contamination (Calafat and Needham 2009). For example, the use of BPA in laboratory supplies necessitates fastidious handling to ensure sample integrity (Ye et al. 2013). Both collection conditions and preservatives to prevent bacterial growth in urine can contribute to contamination of urine samples with BPA (Longnecker et al. 2013). Similarly, personal care products used by laboratory technicians that contain benzophenone-3 and parabens have contaminated biological samples (Ye et al. 2013).

Accurate exposure assessment is critical to establishing reliable estimates of associations between background phenolic exposures and health outcomes. Exposures to select phenols (Meeker et al. 2013; Woodruff et al. 2011; Ye et al. 2009), and the reliability of a single spot urine sample to estimate exposure to phenolic compounds over the course of pregnancy can vary substantially across populations (Braun et al. 2011a, 2012; Jusko et al. 2014; Meeker et al. 2013; Philippat et al. 2013; Quirós-Alcalá et al. 2013). These findings suggest that reliability estimates for these compounds may not be generalizable across populations, particularly when patterns of product usage or population exposures may be different.

The purpose of our study was 3-fold. First, we wanted to measure the concentrations of phenols and parabens in archived urine collected from women in the Norwegian Mother and Child Cohort Study (MoBa). Second, we sought to characterize the reliability of a single spot urine sample to estimate environmental exposure to these compounds over the course of pregnancy in the MoBa cohort. Third, given the recently reported potential for contamination of MoBa specimens (Longnecker et al. 2013), we investigated whether using the free and conjugated concentrations of phenolic biomarkers might improve reliability estimates.

## Methods

*Study design*. MoBa is a prospective population-based pregnancy cohort conducted by the Norwegian Institute of Public Health (Magnus et al. 2006). MoBa was initiated for the study of exposures and diseases related to pregnancy and child development, including potential health effects associated with exposures to environmental chemicals (Magnus et al. 2006). From 1999 through 2008, pregnant women across Norway were recruited at their first ultrasound visit (approximately 17–18 weeks gestation), and 38.5% of invited women consented to participate. The final cohort includes 90,700 mothers and 108,000 pregnancies (Norwegian Institute of Public Health 2013).

Beginning in 2002, as part of the standard data collection protocol, MoBa participants provided a spot urine specimen in a collection cup at the ultrasound visit. Because of evidence of bacterial growth upon receipt at the central processing facility, MoBa revised the protocol to include the addition of a preservative to urine specimens. Laboratory staff transferred 8 mL of urine from each specimen to a Vacutainer tube (Urinalysis Preservative Plus Urine Tube; BD Diagnostics) with a mixture of three preservatives (sodium propionate 94%, ethyl paraben 5.6%, and chlorhexidine 0.4%) and shipped the specimen, unrefrigerated, to a central processing facility (Hoppin et al. 2006; Rønningen et al. 2006). At the processing facility, urine samples were partitioned into 930-μL aliquots and stored at –80°C until use (Rønningen et al. 2006).

From November 2007 through December 2008, a subset of MoBa participants (*n* = 671) donated a spot urine sample at approximately 17 weeks of gestation plus additional urine specimens at approximately 23 and 29 weeks of gestation for a reliability substudy. These participants were recruited from hospitals representing four regions of Norway [northwest (Sunnmøre Hospital HF Ålesund), southwest (Stavanger University Hospital HF), central (St Olavs Hospital HF), and east (Østfold Hospital HF Fredrikstad)] and specimens were collected using the protocol described above.

From the subset of 671, we randomly selected 30 participants for this reliability substudy. Another 15 participants from the same subset of 671 that were part of a previous random selection for an unpublished pilot study were also included. The pilot study showed that these 15 women had the highest measured total BPA concentrations (range, 22.9–52.1 μg/g creatinine); we included them to determine whether the high concentrations could possibly be attributed to contamination. The final study sample thus included 135 urine samples from 45 women.

For quality control (QC), in 2011 we collected spot urine specimens from 10 Norwegian women using the same collection protocol and materials used for MoBa participants, except that the specimens did not require shipping because they were collected from processing facility staff. QC samples were pooled before transfer to the tube with preservative and aliquotted before analysis.

Informed consent was obtained from each MoBa participant on recruitment. The MoBa study was approved by The Regional Committee for Medical Research Ethics in South-Eastern Norway (#2011/1386). The reliability substudy was also approved by The Regional Committee for Medical Research Ethics in South-Eastern Norway and the University of North Carolina Institutional Review Board. The involvement of the CDC laboratory did not constitute engagement in human subjects research.

*Measurement of phenol and paraben concentrations*. In 2012, we analyzed urine specimens for free and total (conjugated plus free) concentrations of nine compounds: BPA, butyl paraben, ethyl paraben, methyl paraben, propyl paraben, benzophenone-3, triclosan, 2,4-dichlorophenol, and 2,5-dichlorophenol. Triclosan results are presented elsewhere (Bertelsen et al. 2014). Analyses were conducted using online solid phase extraction–isotope dilution–high performance liquid chromatography–tandem mass spectrometry (online SPE-HPLC-isotope dilution-MS/MS) (Ye et al. 2005a, 2008) at the CDC (Atlanta, GA, USA) in three analytical batches. All urine specimens from a participant were analyzed in the same analytical batch in random order. The pooled urine for QC was distributed into 15 aliquots, and we included 5 aliquots in each of the three analytical batches. Phenol and paraben concentrations are reported in micrograms per liter and micrograms per gram of creatinine to account for urine dilution.

*Statistical analyses*. All statistical analyses were conducted separately for the complete sample (*n* = 45 participants, 135 samples; hereafter “complete sample”) as well as for the two subgroups: randomly selected participants with no previously measured BPA concentrations (*n* = 30 participants, 90 samples; hereafter “random sample”) and participants with high total BPA concentrations in a previous random selection (*n* = 15 participants, 45 samples; hereafter “high-BPA subgroup”).

We summarized characteristics of the study population using data from version 7 of the quality-assured MoBa data files released for research in June 2012. We also computed coefficients of variation (CVs) for all QC specimens and between the three QC batches. Between-batch CVs based on the QC samples were generally low (< 15%) except when mean concentrations were near the limit of detection (LOD) (see Supplemental Material, Table S1).

For the study sample, we calculated conjugated concentrations as the difference between total and free concentrations, when both total and free concentrations were detectable. Negative conjugated values occurred when free concentrations exceeded total concentrations at levels close to the LOD, which are subject to higher measurement error. We computed geometric means for total, free, and conjugated concentrations with all time periods combined as well as for each of the three time points. We used the instrumental values for total phenol and paraben concentrations below the LOD; for free phenol and paraben concentrations, instrumental values below the LOD were not provided (i.e., missing) because the majority of free concentrations were so low that the instrument software recorded them as “no peak” or “< 0.” We analyzed data only for compounds with > 50% detection frequency; analytes fulfilling this criterion all had 95–100% detection frequency, so we did not employ any additional correction (e.g., imputation) for these values. To examine potential contamination of specimens, we also compared the distributions of free phenol and paraben concentrations as a percentage of the total concentration for the five compounds with sufficient detection: BPA, butyl paraben, methyl paraben, propyl paraben, and benzophenone-3.

We assessed the proportion of variance attributed to between-person variability across the three time points in pregnancy using intraclass correlation coefficients (ICCs). ICCs typically range from 0 to 1 with a value close to 1 indicating high temporal reliability of measurements, with most of the variance attributable to differences between subjects rather than within-person differences between time points. We calculated ICCs for total, free, and conjugated concentrations in micrograms per liter and micrograms per gram creatinine. ICCs and 95% confidence intervals (CIs) were computed with natural logarithm–transformed data using sums of squares from generalized linear models generated by the intracc macro for SAS (Hamer 1995). For comparison, ICCs were also computed using random effects models to estimate within- and between-person variance, assuming an unstructured covariance matrix to allow each covariance to be uniquely estimated and provide maximum model flexibility. The ICCs computed with random-effects models produced results similar to those shown and thus are not presented.

All statistical analyses were conducted using SAS software version 9.3 (SAS Institute Inc., Cary, NC, USA).

## Results

Most MoBa participants were married or cohabitating, had at least some college education, and had a moderate income (Table 1). Although nearly half were ever-smokers, most did not smoke during the current pregnancy. The median age of participants at delivery was 30 years with a range of 20–41 years (random sample: median = 31, range 20–41 years; high-BPA subgroup: median = 29, range 21–37 years). The majority of specimens (79.2%) spent 1 day in transit with the remainder spending 2–5 days.

**Table 1:** Characteristics of complete study sample and subgroups [*n* (%)].*^a^*

Characteristic	Complete sample (*n *= 45)	Random sample (*n *= 30)	High-BPA subgroup (*n *= 15)
Civil status
Married	20 (44.4)	14 (46.7)	6 (40.0)
Cohabitating	21 (46.7)	13 (43.3)	8 (53.3)
Single	2 (4.4)	1 (3.3)	1 (3.3)
Missing	2 (4.4)	2 (6.7)	0 (0.0)
Completed maternal education
< College/university	12 (26.7)	9 (30.0)	3 (20.0)
College/university (up to and including 4 years)	24 (53.3)	16 (53.3)	8 (53.3)
> College/university	6 (13.3)	2 (6.7)	4 (26.7)
Missing	3 (6.7)	3 (10.0)	0 (0.0)
Gross income ($US)
< 34,800	8 (17.8)	5 (16.7)	3 (20.0)
34,800–69,600	27 (60.0)	18 (60.0)	9 (60.0)
> 69,600	7 (15.6)	4 (13.3)	3 (20.0)
Missing	3 (6.7)	3 (10.0)	0 (0.0)
Ever smoked
No	23 (51.1)	16 (53.3)	7 (46.7)
Yes	21 (46.7)	13 (43.3)	8 (53.3)
Missing	1 (2.2)	1 (3.3)	0 (0.0)
Smoked during this pregnancy
No	38 (84.4)	26 (86.7)	12 (80.0)
Sometimes or daily	4 (8.8)	2 (6.7)	2 (13.3)
Missing	3 (6.7)	2 (6.7)	1 (6.7)
^***a***^Complete sample (*n *= 45 participants, 135 samples); random sample: randomly selected participants with no previously measured BPA concentrations (*n *= 30 participants, 90 samples); high-BPA subgroup: participants with high BPA concentrations in a previous random selection (*n *= 15 participants, 45 samples).			

Percent detection was comparable among subgroups but varied by analyte, with most compounds detected in the majority of samples (Tables 2 and 3). We detected free and total concentrations in 100% of samples for four analytes (BPA, butyl paraben, methyl paraben, propyl paraben). We detected total benzophenone-3 in the majority (> 92%) of samples and free benzophenone-3 in approximately 10%. Total concentrations of 2,4-dichlorophenol and 2,5-dichlorophenol were detected in < 50% of samples, with negligible detection of free concentrations for these analytes. Ethyl paraben could not be reliably quantitated because it was a component of the urine preservative added to prevent bacterial growth. Additionally, two samples that did not pass the laboratory QA (quality assurance)/QC criteria for free phenolic compounds concentrations were not reported.

**Table 2:** Limit of detection, percent detectable results, and geometric means for complete study sample and subgroups (μg/L).*^a^*

Analyte and species^*b*^	Complete sample (*n *= 45)			Random sample (*n *= 30)			High-BPA subgroup (*n *= 15)		
	*n* (%) > LOD	GM all time periods	GM 17, 23, 29 weeks	*n* (%) > LOD	GM all time periods	GM 17, 23, 29 weeks	*n* (%) > LOD	GM all time periods	GM 17, 23, 29 weeks
Bisphenol A (LOD = 0.4 μg/L)
Total	135 (100)	7.7	8.9, 7.5, 6.7	90 (100)	7.5	7.8, 7.4, 7.2	45 (100)	8.1	11.6, 7.8, 5.9
Free	133 (100)	5.4	7.2, 5.2, 4.3	89 (100)	5.3	6.0, 5.6, 4.5	44 (100)	5.6	10.5, 4.6, 3.8
Conjugated	133 (100)	1.3	1.1, 1.3, 1.6	89 (100)	1.2	1.0, 1.1, 1.4	44 (100)	1.7	1.5, 1.8, 1.9
Butyl paraben (LOD = 0.2 μg/L)
Total	135 (100)	6.3	6.7, 6.1, 5.9	90 (100)	6.4	7.6, 6.0, 5.7	45 (100)	6.1	5.3, 6.4, 6.5
Free	133 (100)	1.8	1.8, 1.8, 1.8	89 (100)	1.8	1.8, 1.8, 1.9	44 (100)	1.8	1.8, 1.9, 1.8
Conjugated	133 (100)	3.1	3.6, 3.1, 2.8	89 (100)	3.5	4.8, 3.8, 2.5	44 (100)	2.5	2.0, 2.2, 3.3
Methyl paraben (LOD = 1.0 μg/L)
Total	135 (100)	1235.7	1,332, 1,249, 1,134	90 (100)	1213.7	1,297, 1,256, 1,097	45 (100)	1280.9	1,405, 1,234, 1,212
Free	133 (100)	25.7	24.1, 26.4, 26.7	89 (100)	25.7	24.7, 25.2, 27.3	44 (100)	25.7	22.9, 28.9, 25.5
Conjugated	133 (100)	1204.5	1,308, 1,212, 1,104	89 (100)	1181.0	1,271, 1,218, 1,066	44 (100)	1253.5	1,392, 1,202, 1,186
Propyl paraben (LOD = 0.2 μg/L)
Total	135 (100)	32.3	29.5, 37.8, 30.2	90 (100)	36.4	40.1, 38.3, 31.4	45 (100)	25.4	16.1, 36.9, 27.8
Free	133 (100)	1.6	1.4, 1.7, 1.6	89 (100)	1.7	1.6, 1.6, 1.7	44 (100)	1.4	1.1, 1.9, 1.3
Conjugated	133 (100)	28.9	25.5, 35.1, 27.3	89 (100)	33.1	35.7, 36.2, 28.2	44 (100)	22.1	12.3, 33.0, 25.6
Benzophenone‑3 (LOD = 0.4 μg/L)
Total	128 (95)	6.1	4.4, 7.0, 7.3	86 (96)	6.7	5.3, 6.5, 8.7	42 (93)	5.0	3.0, 7.9, 5.2
Free	13 (10)	NC	NC	9 (10)	NC	NC	4 (9)	NC	NC
Conjugated	13 (10)	NC	NC	9 (10)	NC	NC	4 (9)	NC	NC
2,4-DCP (LOD = 0.2 μg/L)
Total	60 (44)	NC	NC	42 (47)	NC	NC	18 (40)	NC	NC
Free	2 (2)	NC	NC	1 (1)	NC	NC	1 (2)	NC	NC
Conjugated	2 (2)	NC	NC	1 (1)	NC	NC	1 (2)	NC	NC
2,5-DCP (LOD = 0.2 μg/L)
Total	19 (14)	NC	NC	11 (12)	NC	NC	8 (18)	NC	NC
Free	0 (0)	NC	NC	0 (0)	NC	NC	0 (0)	NC	NC
Conjugated	0 (0)	NC	NC	0 (0)	NC	NC	0 (0)	NC	NC
Abbreviations: 2,4-DCP, 2,4-dichlorophenol; 2,5-DCP, 2,5-dichlorophenol; GM, geometric mean; LOD, limit of detection; NC, not calculated due to < 50% detection. ^***a***^Complete sample (*n *= 45 participants, 135 samples); random sample: randomly selected participants with no previously measured BPA concentrations (*n *= 30 participants, 90 samples); high-BPA subgroup: participants with high BPA concentrations in a previous random selection (*n *= 15 participants, 45 samples). ^***b***^Instrumental readings used for total concentrations; values > LOD used for free concentrations. Conjugated concentrations, which are the difference between measured total and free concentrations, were calculated if both total and free were > LOD. Two specimens lacked reportable free phenol concentrations due to instrument error.									

**Table 3:** Limit of detection, percent detectable results, and geometric means for complete study sample and subgroups (μg/g creatinine).*^a^*

Analyte and species^*b*^	Complete sample (*n *= 45)			Random sample (*n *= 30)			High-BPA subgroup (*n *= 15)		
	*n* (%) > LOD	GM all time periods	GM 17, 23, 29 weeks	*n* (%) > LOD	GM all time periods	GM 17, 23, 29 weeks	*n* (%) > LOD	GM all time periods	GM 17, 23, 29 weeks
Bisphenol A (LOD = 0.4 μg/L)
Total	135 (100)	10.9	17.9, 9.3, 7.8	90 (100)	9.6	12.3, 9.2, 7.8	45 (100)	14.1	37.8, 9.4, 7.8
Free	133 (100)	7.7	14.3, 6.5, 5.0	89 (100)	6.9	9.5, 7.1, 4.9	44 (100)	9.6	34.1, 5.5, 5.2
Conjugated	133 (100)	1.8	2.1, 1.6, 1.8	89 (100)	1.4	1.5, 1.3, 1.5	44 (100)	2.8	4.5, 2.2, 2.5
Butyl paraben (LOD = 0.2 μg/L)
Total	135 (100)	8.9	13.5, 7.5, 6.9	90 (100)	8.2	12.0, 7.5, 6.1	45 (100)	10.5	17.3, 7.6, 8.9
Free	133 (100)	2.6	3.6, 2.3, 2.1	89 (100)	2.4	2.9, 2.3, 2.0	44 (100)	3.1	5.8, 2.2, 2.4
Conjugated	133 (100)	4.3	6.9, 3.6, 3.3	89 (100)	4.4	7.1, 4.3, 2.7	44 (100)	4.2	6.5, 2.7, 4.5
Methyl paraben (LOD = 1.0 μg/L)
Total	135 (100)	1758.9	2,676, 1,539, 1,322	90 (100)	1563.0	2,051, 1,572, 1,185	45 (100)	2227.5	4,559, 1,474, 1,645
Free	133 (100)	36.6	48.0, 33.0, 31.1	89 (100)	33.3	39.0, 32.2, 29.4	44 (100)	44.2	74.8, 34.5, 34.6
Conjugated	133 (100)	1713.4	2,604, 1,512, 1,286	89 (100)	1530.8	2,010, 1,553, 1,150	44 (100)	2152.1	4,536, 1,435, 1,609
Propyl paraben (LOD = 0.2 μg/L)
Total	135 (100)	46.0	59.3, 46.6, 35.1	90 (100)	46.8	63.3, 47.9, 33.9	45 (100)	46.2	52.1, 44.0, 37.8
Free	133 (100)	2.2	2.8, 2.1, 1.9	89 (100)	2.1	2.5, 2.1, 1.9	44 (100)	2.4	3.6, 2.2, 1.8
Conjugated	133 (100)	41.0	50.7, 43.1, 31.8	89 (100)	42.6	56.4, 45.2, 30.4	44 (100)	38.0	40.2, 39.5, 34.8
Benzophenone‑3 (LOD = 0.4 μg/L)
Total	128 (95)	8.5	8.8, 8.3, 8.5	86 (96)	8.5	8.4, 7.7, 9.4	42 (93)	8.6	9.6, 9.5, 7.0
Free	13 (10)	NC	NC	9 (10)	NC	NC	4 (9)	NC	NC
Conjugated	13 (10)	NC	NC	9 (10)	NC	NC	4 (9)	NC	NC
2,4-DCP (LOD = 0.2 μg/L)
Total	60 (44)	NC	NC	42 (47)	NC	NC	18 (40)	NC	NC
Free	2 (2)	NC	NC	1 (1)	NC	NC	1 (2)	NC	NC
Conjugated	2 (2)	NC	NC	1 (1)	NC	NC	1 (2)	NC	NC
2,5-DCP (LOD = 0.2 μg/L)
Total	19 (14)	NC	NC	11 (12)	NC	NC	8 (18)	NC	NC
Free	0 (0)	NC	NC	0 (0)	NC	NC	0 (0)	NC	NC
Conjugated	0 (0)	NC	NC	0 (0)	NC	NC	0 (0)	NC	NC
Abbreviations: 2,4-DCP, 2,4-dichlorophenol; 2,5-DCP, 2,5-dichlorophenol; GM, geometric mean; LOD, limit of detection; NC, not calculated due to < 50% detection. ^***a***^Complete sample (*n *= 45 participants, 135 samples); random sample: randomly selected participants with no previously measured BPA concentrations (*n *= 30 participants, 90 samples); high-BPA subgroup: participants with high BPA concentrations in a previous random selection (*n *= 15 participants, 45 samples). ^***b***^Instrumental readings used for total concentrations; values > LOD used for free concentrations. Conjugated concentrations, which are the difference between measured total and free concentrations, were calculated if both total and free were > LOD. Two specimens lacked reportable free phenol concentrations due to instrument error.									

The geometric mean concentration of analytes for the random sample and high-BPA subgroup were generally comparable when expressed on a micrograms per liter basis (Table 2). When expressed on a micrograms per gram creatinine basis, however, we observed a higher mean free BPA concentration at week 17 for the high-BPA subgroup (Table 3). In turn, we observed a difference in mean total BPA concentrations between subgroups, as confirmed with a *t*-test comparing log-normally distributed means (data not shown). We also observed a higher mean conjugated methyl paraben for the high-BPA subgroup, which led to a significant difference in mean total methyl paraben concentrations between subgroups (data not shown).

For all phenols and parabens, the percentage of free species in the total concentration was similar for the random sample and high-BPA subgroup (Table 4). The majority of BPA and butyl paraben was in its free form. Free concentration comprised over 20% of the total biomarker concentration in approximately 96% of the samples analyzed for BPA and approximately 65% of the samples analyzed for butyl paraben (Table 4). By contrast, free propyl paraben represented > 20% of the total concentration in approximately 11% of samples, and most of the detected methyl paraben and benzophenone-3 was conjugated.

**Table 4:** Comparison of free concentrations as a percent of total concentration (μg/L) for select phenols and parabens within complete study sample and subgroups [*n* (%)].*^a^*

Percentage of free/total	Bisphenol A			Butyl paraben			Methyl paraben			Propyl paraben			Benzophenone-3		
	Complete sample (*n *= 45)	Random sample (*n *= 30)	High-BPA subgroup (*n *= 15)	Complete sample (*n *= 45)	Random sample (*n *= 30)	High-BPA subgroup (*n *= 15)	Complete sample (*n *= 45)	Random sample (*n *= 30)	High-BPA subgroup (*n *= 15)	Complete sample (*n *= 45)	Random sample (*n *= 30)	High-BPA subgroup (*n *= 15)	Complete sample (*n *= 45)	Random sample (*n *= 30)	High-BPA subgroup (*n *= 15)
Missing	2 (1.5)	1 (1.1)	1 (2.2)	2 (1.5)	1 (1.1)	1 (2.2)	2 (1.5)	1 (1.1)	1 (2.2)	2 (1.5)	1 (1.1)	1 (2.2)	2 (1.5)	1 (1.1)	1 (2.2)
Free < LOD	0 (0.0)	0 (0.0)	0 (0.0)	0 (0.0)	0 (0.0)	0 (0.0)	0 (0.0)	0 (0.0)	0 (0.0)	0 (0.0)	0 (0.0)	0 (0.0)	120 (88.9)	80 (88.9)	40 (88.9)
0–5%	0 (0.0)	0 (0.0)	0 (0.0)	6 (4.4)	5 (5.6)	1 (2.2)	130 (96.3)	87 (96.7)	43 (95.6)	71 (52.6)	51 (56.7)	20 (44.4)	9 (6.7)	6 (6.7)	3 (6.7)
> 5–10%	1 (0.7)	1 (1.1)	0 (0.0)	18 (13.3)	10 (11.1)	8 (17.8)	2 (1.5)	1 (1.1)	1 (2.2)	31 (23.0)	17 (18.9)	14 (31.1)	2 (1.5)	2 (2.2)	0 (0.0)
> 10–20%	2 (1.5)	1 (1.1)	1 (2.2)	21 (15.6)	16 (17.8)	5 (11.1)	1 (0.7)	1 (1.1)	0 (0.0)	16 (11.9)	12 (13.3)	4 (8.9)	1 (0.7)	1 (1.1)	0 (0.0)
> 20–50%	16 (11.9)	13 (14.4)	3 (6.7)	38 (28.2)	28 (31.1)	10 (22.2)	0 (0.0)	0 (0.0)	0 (0.0)	7 (5.2)	3 (3.3)	4 (8.9)	1 (0.7)	0 (0.0)	1 (2.2)
> 50%	114 (84.4)	74 (82.2)	40 (88.9)	50 (37.0)	30 (33.3)	20 (44.4)	0 (0.0)	0 (0.0)	0 (0.0)	8 (5.9)	6 (6.7)	2 (4.4)	0 (0.0)	0 (0.0)	0 (0.0)
LOD, limit of detection. ^***a***^Complete sample (*n *= 45 participants, 135 samples); random sample: randomly selected participants with no previously measured BPA concentrations (*n *= 30 participants, 90 samples); high-BPA subgroup: participants with high BPA concentrations in a previous random selection (*n *= 15 participants, 45 samples).															

For the conjugated concentrations of BPA, butyl paraben, and propyl paraben (Table 5), the ICCs ranged from low for BPA (~ 0.25) to moderate for butyl paraben (~ 0.40) and propyl paraben (~ 0.60); this was the case regardless of subgroup or method of expressing concentration. For conjugated methyl paraben, the ICCs varied both by subgroup and method of expressing concentration, with values ranging from moderate to low—for example, 0.34 in micrograms per gram creatinine for the random sample and 0.06 in micrograms per gram creatinine for the high-BPA subgroup.

**Table 5:** ICCs (95% CIs) for total, conjugated, and free concentrations of phenols and parabens for complete study sample and subgroups.*^a^*

Analyte	Unadjusted concentration (μg/L)			Adjusted concentration (μg/g creatinine)		
	Total	Conjugated	Free	Total	Conjugated	Free
Complete sample (*n *= 45)
BPA	0.24 (0.09, 0.39)	0.29 (0.13, 0.44)	0.15 (0.01, 0.29)	0.04 (–0.07, 0.17)	0.24 (0.09, 0.39)	0.02 (–0.09, 0.14)
Butyl paraben	0.34 (0.18, 0.49)	0.36 (0.20, 0.50)	0.10 (–0.04, 0.26)	0.38 (0.23, 0.53)	0.41 (0.26, 0.55)	0.12 (–0.01, 0.26)
Methyl paraben	0.48 (0.33, 0.61)	0.47 (0.33, 0.61)	0.34 (0.18, 0.48)	0.24 (0.10, 0.40)	0.24 (0.09, 0.39)	0.24 (0.10, 0.39)
Propyl paraben	0.55 (0.41, 0.67)	0.57 (0.44, 0.69)	0.56 (0.42, 0.68)	0.62 (0.49, 0.72)	0.64 (0.51, 0.74)	0.61 (0.47, 0.72)
Benzophenone-3	0.43 (0.28, 0.57)	NC	NC	0.50 (0.35, 0.63)	NC	NC
Random sample (*n *= 30)
BPA	0.36 (0.20, 0.50)	0.28 (0.13, 0.43)	0.24 (0.09, 0.40)	0.13 (–0.01, 0.29)	0.15 (0.00, 0.31)	0.11 (–0.02, 0.26)
Butyl paraben	0.27 (0.12, 0.43)	0.34 (0.19, 0.49)	0.11 (–0.04, 0.26)	0.30 (0.16, 0.46)	0.36 (0.21, 0.51)	0.22 (0.08, 0.38)
Methyl paraben	0.53 (0.39, 0.66)	0.52 (0.39, 0.65)	0.34 (0.18, 0.48)	0.35 (0.21, 0.50)	0.34 (0.20, 0.50)	0.24 (0.10, 0.40)
Propyl paraben	0.51 (0.37, 0.64)	0.53 (0.39, 0.65)	0.57 (0.43, 0.69)	0.58 (0.45, 0.70)	0.59 (0.47, 0.71)	0.71 (0.61, 0.80)
Benzophenone-3	0.38 (0.23, 0.52)	NC	NC	0.38 (0.23, 0.53)	NC	NC
High BPA sample (*n *= 15)
BPA^*b*^	0.01 (–0.11, 0.14)	0.22 (0.07, 0.37)	0.00 (–0.10, 0.13)	–0.12 (–0.25, –0.12)	0.18 (0.05, 0.35)	–0.11 (–0.23, –0.07)
Butyl paraben^*b*^	0.50 (0.34, 0.62)	0.42 (0.26, 0.55)	0.11 (–0.03, 0.27)	0.54 (0.44, 0.69)	0.54 (0.42, 0.67)	–0.05 (–0.16, 0.05)
Methyl paraben	0.36 (0.21, 0.51)	0.35 (0.20, 0.50)	0.37 (0.23, 0.52)	0.06 (–0.03, 0.22)	0.06 (–0.04, 0.21)	0.21 (0.10, 0.39)
Propyl paraben	0.66 (0.56, 0.77)	0.67 (0.58, 0.78)	0.54 (0.43, 0.68)	0.73 (0.62, 0.81)	0.75 (0.65, 0.82)	0.47 (0.35, 0.63)
Benzophenone-3	0.59 (0.50, 0.74)	NC	NC	0.80 (0.71, 0.86)	NC	NC
NC, not calculated due to < 50% detection. ^***a***^Complete sample (*n *= 45 participants, 135 samples); random sample: randomly selected participants with no previously measured BPA concentrations (*n *= 30 participants, 90 samples); high-BPA subgroup: participants with high BPA concentrations in a previous random selection (*n *= 15 participants, 45 samples). ^***b***^When the variance within time points exceeds the variance between subjects, a negative ICC is mathematically possible and can be interpreted as approximately 0 (Shrout and Fleiss 1979).						

For the free concentrations of BPA and butyl paraben, the ICCs were lower than for the conjugated concentrations, even approaching 0 for free BPA. This was true regardless of method of expressing concentration or subgroup. The ICC for free methyl paraben was approximately 0.35 across subgroups in micrograms per liter and approximately 0.2 in micrograms per gram creatinine. For creatinine-adjusted free propyl paraben, the ICC was higher in the random sample (0.71) than the high-BPA subgroup (0.47), whereas unadjusted values were more consistent across subgroups (~ 0.55).

The ICC for the total concentrations of these phenols and parabens can be thought of as a weighted average of the values for the conjugated and free compounds. Thus, for BPA and butyl paraben, with a relatively greater proportion of free compound, the ICCs for total concentrations were generally low. For methyl paraben and propyl paraben, with relatively little free compound, the ICCs for total concentrations largely reflected the ICCs of conjugated concentrations. For BP-3, where a lack of detectable free concentrations prevented the computation of conjugated concentrations, the ICC was 0.38 in the random sample and 0.80 in the high-BPA subgroup.

ICCs computed with random effects models produced similar results (data not shown). The Spearman correlation coefficients among serial pairs of concentrations (conjugated, creatinine-adjusted) were generally comparable across pairing and subgroups, consistent with the results in Table 5 (see Supplemental Material, Table S2).

## Discussion

The main purpose of our study was to examine the reliability of phenolic concentrations in a single spot urine sample collected during pregnancy to estimate exposure over the course of pregnancy in the MoBa cohort. In general, ICCs were poor to moderate, with the highest ICCs found consistently for propyl paraben (~ 0.60). We expect ICCs for concentrations that have been adjusted for creatinine to more accurately describe exposure variability, rather than variability in urine dilution. Results were generally similar overall and by subgroup. The small sample size (*n* = 45) may also have contributed to imprecision in the estimates of average concentration and of the ICCs.

Contamination likely accounts for the greater geometric mean total BPA concentrations in this study sample compared with other studies. When free BPA is excluded from the total analyte concentration, the geometric mean for conjugated BPA (1.3 μg/L) is similar to other population estimates for total BPA (Braun et al. 2009; Bushnik et al. 2010; CDC 2013; Hoepner et al. 2013), including those for pregnant women (Quirós-Alcalá et al. 2013), although some previously reported total BPA means for pregnant women have been higher than these conjugated values (Meeker et al. 2013; Woodruff et al. 2011). Quantifying free BPA only provides an estimate of potential contamination. For butyl paraben, 88 (65%) samples had free concentrations greater than 20% of total concentrations, also indicating likely contamination. There was some evidence of contamination with propyl paraben as well; for propyl paraben, 15 (11%) samples had free concentrations > 20% of total, but reliability estimates were consistent for total, conjugated, and free concentrations. There was no evidence of contamination for methyl paraben, benzophenone-3, 2,4-dichlorophenol, or 2,5-dichlorophenol, given either the limited proportion of free concentrations in the total concentration (methyl paraben, benzophenone-3) or the limited detection of any free concentrations among those samples with detectable total concentrations (2,4-dichlorophenol, 2,5-dichlorophenol).

In the case of MoBa, the likely sources of BPA contamination were the urinary preservative (sodium propionate 94%, ethyl paraben 5.6%, and chlorhexidine 0.4%) and the collection conditions, that is, the hospital setting, plastic materials, and handling procedures (Longnecker et al. 2013), despite employing procedures to limit possible contamination. Population sources of BPA exposure are diverse. BPA is employed in the manufacture of polycarbonate plastics (e.g., compact discs, plastic dinnerware, toys), epoxy resins (e.g., can linings, dental composites), and thermal paper (e.g., some receipts) (CDC 2009). Our results suggest that the magnitude of contamination varied, perhaps due to collection conditions or changes in preservative composition, and thus measurement of free concentrations would be required to isolate the possible contaminant from the conjugated fraction. Many investigators take steps to reduce contamination from known sources; however, contamination may also arise in unexpected ways. It is difficult to compare possible sources of contamination resulting from sample collection procedures between studies due to limited detail provided in the peer-reviewed literature. Although most investigators choose to measure only total phenol and paraben concentrations (conjugated and free combined), measuring both species in at least a subgroup of study samples allows for the identification of samples in which the free:total proportion is out of the expected range (Koch et al. 2012; Völkel et al. 2002; Waechter et al. 2007), which may indicate contamination.

We also investigated whether isolating the conjugated concentrations would produce improved ICCs compared with those for total concentrations. Separating the conjugated from the free phenol did not substantially alter reliability estimates for most analytes. For both BPA and butyl paraben; however, the majority of samples had a substantial proportion of these compounds present as free species and the conjugated analyte concentrations appeared more reliable, whereas ICCs for free concentrations were especially low. This supports four conclusions: *a*) Reliability estimates may be affected by external sources of contamination, as indicated by higher concentrations than typically reported in other studies; *b*) in this population, the extent of the specimen contamination varied, possibly due to conditions at multiple collection locations or variation in collection tube manufacturing (Table 2); *c*) these data may also reflect daily variability in individual exposures that make characterization of average longer-term exposure challenging; and *d*) the reliability of a single specimen’s concentration to categorize BPA exposure is rather poor, as has been reported in other studies (Braun et al. 2011a, 2012; Jusko et al. 2014; Meeker et al. 2013; Philippat et al. 2013). For other phenolic compounds, our results were comparable to previous estimates for methyl paraben, but lower than prior reports for benzophenone-3, butyl paraben, and 2,4-dichlorophenol, and higher than prior reports for propyl paraben (Meeker et al. 2013; Philippat et al. 2013; Smith et al. 2012).

Although the source of butyl paraben contamination in the MoBa samples could not be definitively established with the available information, it appears that the urinary preservative containing 5.6% ethyl paraben was the source of the detected ethyl paraben. The analytic consequences of this preservative are of primary importance to investigators analyzing affected specimens. Online SPE-HPLC-isotope dilution-MS/MS includes three steps: an extraction step in which target analytes are preconcentrated and separated from unwanted matrix components, a separation step in which target compounds are separated from each other and residual matrix biomolecules, and a detection step in which compounds are quantified according to molecular mass. Generally, environmental chemicals, including phenols and parabens, are present in urine at trace concentrations—at or below parts per billion. In the MoBA samples, ethyl paraben concentrations were in the parts per thousand range. Therefore, the detector was oversaturated, and the analytic performance and sensitivity of the mass spectrometer for all phenols and parabens measured was negatively affected, as evidenced by the QC CVs being in some cases twice as large as annual CVs reported by the CDC for these analytes (CDC, National Center for Environmental Health 2011).

Given the extent of interference, exposure assessment for most urinary phenols and parabens may be difficult in the MoBa cohort using the currently available assays. It is unknown to what degree additional analytes might be impacted. We hypothesize that the extraction, separation, or quantification of compounds with physicochemical properties comparable to those of ethyl paraben may also be negatively impacted. Further detailed quality control studies would have to be conducted in order to test this hypothesis.

## Conclusions

We demonstrated moderate reliability of a single spot urinary concentration to estimate exposure over an 18-week period for benzophenone-3 and propyl paraben, with lower reliability for the other measured analytes. Studies interested in measuring environmental chemicals should avoid, if at all possible, the use of preservatives that may interfere with analytic procedures, and should provide collection protocols to the analytic laboratory in advance so potential interferences can be anticipated. We document the utility of measuring the total and free phenol and paraben biomarker concentrations as a method for detecting the contamination of specimens from these ubiquitous compounds.

## Supplemental Material

(176 KB) PDF
