# Supplementary material for: Measurement of Total and Free Urinary Phenol and Paraben Concentrations over the Course of Pregnancy: Assessing Reliability and Contamination of Specimens in the Norwegian Mother and Child Cohort Study
Source: Environ Health Perspect. 2015 Mar 17;123(7):705–11. doi: 10.1289/ehp.1408325 (PMC4492266; doi:10.1289/ehp.1408325)
Supplement: (176 KB) PDF [file ehp.1408325.s001.acco.pdf]

*Environ Health Perspect*
doi: 10.1289/ehp.1408325

**Note to Readers: *EHP* strives to ensure that all journal content is accessible to all readers. However, some figures and Supplemental Material published in *EHP* articles may not conform to 508 standards due to the complexity of the information being presented. If you need assistance accessing journal content, please contact ehp508@niehs.nih.gov. Our staff will work with you to assess and meet your accessibility needs within 3 working days.**

## Supplemental Material

# Measurement of Total and Free Urinary Phenol and Paraben Concentrations over the Course of Pregnancy: Assessing Reliability and Contamination of Specimens in the Norwegian Mother and Child Cohort Study

Virginia T. Guidry, Matthew P. Longnecker, Heidi Aase, Merete Eggesbø, Pål Zeiner, Ted Reichborn-Kjennerud, Gun P. Knudsen, Randi J. Bertelsen, Xiaoyun Ye, Antonia M. Calafat, Stephanie M. Engel

## Table of Contents

**Table S1.** Limit of detection (LOD), number and percent >LOD, geometric means, and coefficient of variation for quality control samples (n=15 aliquots from common urinary pool of 10 women).

**Table S2.** Spearman pairwise correlation coefficients for conjugated concentrations of phenols and parabens in micrograms per gram creatinine for complete study sample and subgroups.

**Table S1.** Limit of detection (LOD), number and percent >LOD, geometric means, and coefficient of variation for quality control samples (n=15 aliquots from common urinary pool of 10 women).[a]

| Analyte | LOD (µg/L) | Species | N (%) >LOD | GM (µg/L) | CV (%) | Between-batch CV (%) |
|---|---|---|---|---|---|---|
| Bisphenol-A | 0.4 | Total | 15 (100) | 8.6 | 7.9 | <5% |
| | | Free | 15 (100) | 6.9 | 6.2 | <5% |
| Butyl Paraben | 0.2 | Total | 15 (100) | 4.2 | 7.0 | <5% |
| | | Free | 15 (100) | 2.9 | 8.0 | <5% |
| Methyl Paraben | 1.0 | Total | 15 (100) | 862.6 | 12.8 | 10.2 |
| | | Free | 15 (100) | 14.3 | 8.7 | <5% |
| Propyl Paraben | 0.2 | Total | 15 (100) | 8.0 | 6.0 | <5% |
| | | Free | 15 (100) | 0.3 | 24.2 | <5% |
| Benzophenone-3 | 0.4 | Total | 15 (100) | 46.1 | 11.3 | <5% |
| | | Free | 2 (13) | NC | NC | NC |
| 2,4-Dichlorophenol | 0.2 | Total | 15 (100) | 0.3 | 24.5 | <5% |
| | | Free | 2 (13) | NC | NC | NC |
| 2,5-Dichlorophenol | 0.2 | Total | 2 (13) | NC | NC | NC |
| | | Free | 0 ( 0) | NC | NC | NC |

LOD = Limit of detection; µg/L = micrograms per liter; GM = Geometric mean; CV = Coefficient of variation; NC = Not calculated due to insufficient sample size.

[a]Instrumental readings used for calculations with total concentrations; only values >LOD used for free fraction (instrumental readings below LOD not available). Quality control samples analyzed in three batches during a three week period.

**Table S2.** Spearman pairwise correlation coefficients for conjugated concentrations of phenols and parabens in micrograms per gram creatinine for complete study sample and subgroups.[a]

| | Complete sample (n=45) | | | Random Sample (n=30) | | | High-BPA Subgroup (n=15) | | |
|---|---|---|---|---|---|---|---|---|---|
| **Analyte** | **17 & 23 weeks** | **23 & 29 weeks** | **17 & 29 weeks** | **17 & 23 weeks** | **23 & 29 weeks** | **17 & 29 weeks** | **17 & 23 weeks** | **23 & 29 weeks** | **17 & 29 weeks** |
| BPA | 0.32 | 0.30 | 0.34 | 0.29 | 0.18 | 0.29 | 0.25 | 0.43 | 0.40 |
| Butyl paraben | 0.39 | 0.43 | 0.41 | 0.24 | 0.45 | 0.38 | 0.66 | 0.49 | 0.56 |
| Methyl paraben | 0.17 | 0.42 | 0.19 | 0.20 | 0.61 | 0.25 | 0.32 | 0.16 | 0.27 |
| Propyl paraben | 0.56 | 0.65 | 0.62 | 0.51 | 0.62 | 0.62 | 0.68 | 0.69 | 0.65 |
| BP-3[b] | 0.75 | 0.62 | 0.53 | 0.65 | 0.48 | 0.42 | 0.90 | 0.90 | 0.79 |

BPA = Bisphenol-A; BP-3 = Benzophenone-3

[a]Complete Sample (n = 45 participants, 135 samples); Random Sample = randomly selected participants with no previously measured BPA concentrations (n = 30 participants, 90 samples); High-BPA Subgroup = participants with high BPA concentrations in a previous random selection (n = 15 participants, 45 samples). [b]Total concentrations (rather than conjugated) used for comparison because 89% of samples had free concentrations below the detection limit, precluding the computation of conjugated concentrations.
